# A Comparative Study on Calprotectin Concentration in Periodontitis Patients Before and After Non-surgical Periodontal Therapy

**DOI:** 10.7759/cureus.78221

**Published:** 2025-01-30

**Authors:** Pallavi Menon, Lakshmi Puzhankara, Archana Venugopal, Shilpa Ramachandran

**Affiliations:** 1 Department of Periodontics, Amrita School of Dentistry, Amrita Vishwa Vidyapeetham, Kochi, IND; 2 Department of Periodontology, Manipal College of Dental Sciences, Manipal, IND; 3 Department of Periodontics, Al-Azhar Dental College, Thodupuzha, IND

**Keywords:** biomarker, calprotectin, gingival crevicular fluid, inflammatory mediator, non-surgical periodontal therapy, periodontitis, saliva, serum

## Abstract

Introduction

Numerous inflammatory mediators exist in body secretions like gingival crevicular fluid (GCF) during periodontitis. One such inflammatory mediator is calprotectin, a protein which is released from leukocytes and other inflammatory cells.

Aims

This study aimed to compare the concentration of calprotectin in the GCF of individuals with and without periodontitis, as well as in the GCF, serum, and saliva of periodontitis patients before and after non-surgical periodontal therapy.

Materials and methods

Subjects were categorized into two groups: Group A included 40 healthy subjects without periodontitis and Group B included 40 subjects with stage 2 grade B periodontitis subjects. Clinical parameters along with calprotectin concentration in both groups were recorded at baseline and compared. All subjects in Group B received non-surgical periodontal therapy (NSPT). Three months following therapy, periodontal parameters and calprotectin concentrations in GCF, serum, and saliva were re-assessed, and a comparison was made with the values obtained before therapy.

Results

At baseline, inter-group comparison revealed that the calprotectin levels in GCF of Group A subjects were significantly lower than Group B subjects. On intra-group comparison within Group B subjects, three months following NSPT, there was a reduction in the concentration of calprotectin in GCF, which was statistically significant.

Conclusion

Calprotectin in GCF can serve as a diagnostic biomarker and maybe used to assess the response to treatment.

## Introduction

Periodontitis, by definition, is “an inflammatory disease of the supporting tissues of the teeth caused by specific micro-organisms or groups of specific micro-organisms, resulting in progressive destruction of the periodontal ligament and alveolar bone with increased probing depth formation, recession, or both” [1].

Commonly used parameters for the diagnosis of periodontal diseases in clinical practice mostly measure the tissue damage (clinical attachment loss along with loss of bone) that happened previously, which gives information only about the disease severity. Methods for diagnosis should preferably provide information concerning the current status of disease activity, instead of the previous status of a disease, thereby highlighting the importance of biomarkers that indicate the presence or absence of active disease. Numerous biological mediators of inflammation have been identified in tissues aﬀected by periodontitis like, pro-inflammatory mediators and acute-phase proteins. Hence, they are considered as important markers for disease progression [2].

Gingival crevicular ﬂuid (GCF) has proven to be a source of various biomarkers and can be collected non-invasively from areas in close proximity to tissues that have been affected by periodontal disease [3].

During inflammation, neutrophils, monocytes, and macrophages get activated along with some specific epithelial cells, and secrete calprotectin, which is also known as myeloid-related protein (MRP8/14) and S100A8/A9 [4], and is an authenticated biomarker in faeces of patients with certain inﬂammatory bowel diseases [5]. High levels of calprotectin were found in blood drawn from rheumatoid arthritis patients [6] and in cases of certain infections and febrile conditions [7]. Calprotectin in GCF was found by Anderson et al. (1994) [8] among subjects with periodontal inflammation. Many other studies have pointed an association between calprotectin in GCF and periodontal parameters [9-12].

Studies that have assessed the influence of non-surgical periodontal therapy (NSPT) on calprotectin levels among chronic periodontitis subjects are limited. Giannopoulou C et al. in 2006 evaluated the calprotectin levels in periodontitis subjects and found that calprotectin significantly reduced 10 days following non-surgical periodontal therapy accompanied by antibiotics that were administered systemically [13]. Studies that have evaluated calprotectin concentration in serum and saliva in those individuals suffering from chronic periodontitis after scaling and root planing alone are also limited.

 Hence, we conducted the current study to assess the capability of calprotectin as an appropriate diagnostic biomarker of inflammation of the periodontium, by assessing whether calprotectin levels can be used to distinguish subjects with and without periodontitis and also by evaluating the influence of NSPT on its concentration.

## Materials and methods

Study population

This research has been performed on patients visiting the Department of Periodontology in Amrita School of Dentistry, Amrita Institute of Medical Sciences (Amrita Vishwa Vidyapeetham), Kochi, Kerala.

The study followed the protocol given by the Declaration of Helsinki and was approved by the Ethical Committee of the Institute (approval no. IEC-AIMS-2017-DENT-194). This research was done over a period between the 10th of June 2017 and the 15th of November 2018.

Subjects were recruited into the study by selecting participants pertaining to the inclusion/exclusion criteria after receiving informed consent from the subjects. After subject selection, they were categorized as Group A (subjects not presenting with periodontitis) and Group B (subjects diagnosed with stage 2 grade B periodontitis) (on the basis of classification by World Workshop 2017 Classification of Periodontal and Peri-Implant Diseases and Conditions).

Systemically healthy subjects falling into the age group of 30-65 years and having a minimum presence of 20 natural teeth were enrolled. Group A included subjects having no periodontitis, no sites with probing depth more than 3 mm, without any clinical attachment loss, and in Group B, subjects having generalized Stage 2 Grade B periodontitis as defined by interdental clinical attachment loss 3-4 mm (at the site of the greatest loss), probing a depth of 4-5 mm involving more than 30% teeth with a moderate rate of progression, were enrolled.

Patients excluded were those with any systemic conditions, pregnant women/lactating mothers, those with any infections, subjects under antibiotic therapy, steroids or treatment for diseases affecting the periodontium for the past six months, and patients with the habit of chewing/smoking tobacco.

Forty subjects per group were necessary to provide a 95% confidence interval and 80% power, based on data from a previously published study by Andersen et al. [3].

Participants of this research signed a written informed consent before the commencement of the study.

Study design

At baseline, to assess the status of oral hygiene, the Simplified Oral Hygiene Index (OHI-S) was used, which was developed by John C. Green and Jack R. Vermillion in 1964 [14]. It has two components, the Debris Index-Simplified (DI-S) and the Calculus Index-Simplified (CI-S), which are added together for the OHI-S score. Its value ranges from 0 to 6, which can be interpreted as good (0.0 to 1.2), fair (1.3 to 3.0), and poor (3.1 to 6).

The severity of gingival inflammation was recorded by assessing the gingival index (GI), which was developed by Loe H and Silness J in 1963 [15]. This index assesses the severity of gingivitis and its location in four possible areas by examining all surfaces of all teeth. Score 0 indicates the absence of inflammation/normal gingiva; 1: mild inflammation, a slight change in color, slight edema, no bleeding on probing; 2: moderate inflammation; moderate glazing, redness, edema, and hypertrophy, bleeding on probing; 3: severe inflammation; marked redness and hypertrophy ulceration, the tendency for spontaneous bleeding.

Parameters used to measure periodontal tissue destruction were the pocket probing depth (PPD) and clinical attachment level (CAL) using the University of Michigan “O” probe with William’s markings by six-point probing, excluding the third molars. A customized acrylic stent with a guiding groove was fabricated for these measurements, for every subject.

GCF, salivary, and serum calprotectin levels were evaluated at baseline. GCF was collected into Eppendorf tubes from the deepest pocket in each subject using a micropipette, stimulated saliva collection was done by instructing the subjects to chew softened paraffin wax for one minute, and blood was collected into Eppendorf tubes from the antecubital vein using 10 ml syringe with the needle size of 0.55 × 25 mm. All samples were stored at -20℃. Calprotectin concentrations in GCF, saliva, and serum were estimated with the help of an ELISA kit. 

At baseline, after recording all the parameters, all participants received instructions to maintain proper oral hygiene and NSPT, which comprised supragingival scaling, subgingival scaling, and root planing.

Three months following NSPT, GCF, salivary, and serum calprotectin levels were recorded again during the recall visit along with clinical periodontal parameters.

Statistical analysis

A statistical software (IBM SPSS Statistics for Windows, Version 20.0 (released 2011, IBM Corp., Armonk, NY)) was used to perform the statistical analysis. Levene’s test was performed to check for equality of variance.

Statistical analysis of the inter-group difference in the clinical parameters and calprotectin concentration between Group A and B was done using an independent sample t-test and to analyze the change in the parameters assessed clinically and calprotectin level in GCF, saliva, and serum of chronic periodontitis subjects from baseline to three months period (intragroup comparison). A paired ‘t’ test was used. All continuous variables were denoted as mean ± standard deviation (SD).

A p-value <0.05 was set to be the level of significance.

## Results

This study included 22 males and 19 females in Group A and 21 males and 19 females in Group B. The mean age (in years) of subjects in Group A was 34.6 ± 2.0 and in Group B was 47.8 ± 8.0.

Table 1 shows a comparison of clinical periodontal parameters, i.e., GI, OHI-S, PPD and CAL between Groups A and B. The difference in the baseline clinical parameters on the inter-group comparison was statistically significant.

**Table 1:** Comparison of clinical periodontal parameters at baseline between healthy subjects and subjects with periodontitis GCF: gingival crevicular fluid; n: total number of subjects; SD: standard deviation; GI: gingival index; OHI-S: Simplified Oral Hygiene Index; PPD: probing pocket depth; mm: millimeter; CAL: clinical attachment loss *Statistically significant at p < 0.05; Independent sample ‘t’-test

Variable (at baseline)	Groups				p-value
	Group A (Healthy) (n = 40)		Group B (Periodontitis) (n = 40)		
	Mean	SD	Mean	SD	
GI	0.40	0.17	0.61	0.35	0.007*
OHI-S	0.51	0.35	0.74	0.38	0.004*
PPD (in mm)	1.87	0.33	3.32	0.47	<0.001*
CAL (in mm)	1.77	0.42	3.37	0.49	<0.001*

Table 2 shows a comparison of calprotectin concentration in GCF (in μg/μl) between Groups A and B. The mean GCF calprotectin concentration at baseline of Group A was 0.04 + 0.05 and Group B subjects was 0.30 + 0.08, thereby exhibiting a difference that was statistically significant (p-value < 0.001).

**Table 2:** Comparison of calprotectin levels in GCF at baseline between healthy subjects and subjects with periodontitis GCF: gingival crevicular fluid; n: total number of subjects; SD: standard deviation; μg/μl: microgram per microliter *Statistically significant at p < 0.05; Independent sample ‘t’-test

Variable (at baseline)	Groupd				p-value
	Group A (Healthy) (n = 40)		Group B (Periodontitis) (n = 40)		
	Mean	SD	Mean	SD	
GCF calprotectin (in μg/μl)	0.04	0.05	0.30	0.08	<0.001*

Table 3 demonstrates the assessment of clinical parameters, i.e., GI, OHI-S, PPD, and CAL in Group B subjects at baseline and three months following NSPT. On intragroup comparison, the difference in all clinical parameters at baseline and three months following NSPT showed a difference that was statistically significant.

**Table 3:** Comparison of clinical periodontal parameters of periodontitis subjects between baseline and three months after non-surgical periodontal therapy (NSPT) NSPT: non-surgical periodontal therapy; SD: standard deviation; n: total number of subjects; GI: gingival index; OHI-S: Simplified Oral Hygiene Index; PPD: probing pocket depth; mm: millimeter; CAL: clinical attachment loss *Statistically significant at p < 0.05; Paired ‘t’ test

Variable	Group B - Periodontitis Group (n =40)				p-value
	At baseline		Three months after NSPT		
	Mean	SD	Mean	SD	
GI	0.61	0.35	0.45	0.28	<0.001*
OHI-S	0.61	0.35	0.45	0.28	<0.001*
PPD(in mm)	3.31	0.47	2.57	0.55	<0.001*
CAL(in mm)	3.37	0.49	2.60	0.60	<0.001*

Table 4 shows a comparative evaluation of the concentration of calprotectin in GCF, saliva, and serum in Group B subjects at baseline and three months following NSPT. The mean value of concentration of calprotectin in GCF at baseline of Group B subjects was 0.31+0.08 and three months after NSPT was 0.16 + 0.09. Hence, there was a difference that was statistically significant (p-value < 0.001). The difference in the mean calprotectin concentration in saliva, from baseline values among Periodontitis subjects (0.17 + 0.12) to three months after NSPT (0.10 + 0.09) and in the mean serum calprotectin concentration at baseline of the periodontitis subjects (0.20 + 0.11) and three months after NSPT (0.20 + 0.10) was not statistically significant.

**Table 4:** Comparison of calprotectin concentration in GCF, saliva, and serum of chronic periodontitis subjects between baseline and three months after non-surgical periodontal therapy (NSPT) NSPT: non-surgical periodontal therapy; SD: standard deviation; GCF: gingival crevicular fluid; n: total number of subjects; μg/μl: microgram per microliter *Statistically significant at p < 0.05; Paired ‘t’ test

Variable	Group B - Periodontitis Group (n =40)				p-value
	At Baseline		3 months after NSPT		
	Mean	SD	Mean	SD	
GCF calprotectin (in μg/μl)	0.31	0.08	0.16	0.09	<0.001*
Salivary calprotectin (in μg/μl)	0.17	0.12	0.10	0.09	0.094
Serum calprotectin (in μg/μl)	0.20	0.11	0.20	0.10	0.947

## Discussion

Calprotectin is a protein found mostly in neutrophils constituting 45% of the total protein content [11]. It is made up of a light and a heavy chain, 11 kDa and 14 kDa, respectively.

In response to bacteria, an inflammatory process is initiated within the periodontium, which begins as gingivitis with an enhanced permeability of the blood vessels permitting the inflow of leukocytes from the gingival vasculature to the crevice, following which T and B cells come into action along with various other inflammatory mediators [16].

Calprotectin is secreted following the death or activation of inflammatory cells. There have been studies that have identified the presence of calprotectin in GCF and those that have shown that calprotectin levels correspond to other markers of periodontal disease [12,17].

This research was performed to compare the calprotectin concentration in GCF of subjects who are free from periodontitis and those subjects diagnosed with chronic moderate periodontitis and to compare the concentration of calprotectin in GCF, saliva, and serum before and after NSPT in subjects with chronic moderate periodontitis.

In spite of the fact that pocket probing depth is not an ideal indicator of the present disease activity, it is commonly utilized to estimate the intensity of periodontitis. Results of this study indicate that the periodontal parameters assessed clinically namely GI, OHI-S, PPD, and CAL showed greater values in the individuals having periodontitis than the subjects without periodontitis, which was of statistical significance.

The results show that the mean value of the concentration of calprotectin in the GCF of subjects without periodontitis was 0.04 μg/μl and that of chronic moderate periodontitis subjects was 0.30 μg/μl, showing a statistically significant difference, which is in accordance with the study by Kido et al. [9] and Anderson et al. [3].

In our study, clinical parameters, i.e., OHI-S, GI, PPD, and CAL, showed statistically significant reduction indicating a reduction in the periodontal inflammation following SRP, which was similar to the results shown in the articles by Badersten et al. [18] and Obeid et al. [19], emphasizing the role of scaling and root planing in improving the status of periodontium in subjects with periodontitis. Hence, it was anticipated that SRP could possibly lead to a reduction in the concentration of calprotectin in GCF, saliva, and serum, although periodontal pocket elimination and gain in clinical attachment loss may not be achieved completely, because SRP alone cannot eliminate the tissue-invading pathogens.

Our results show that the concentration of calprotectin in GCF of periodontitis subjects at baseline was 0.31 μg/μl and at three months after NSPT was 0.16 μg/μl. Hence, a reduction that was statistically significant was seen in the concentration of calprotectin in GCF from baseline to three months following NSPT, which is consistent with the research of Anderson et al. [3], Gao et al. [20], and Mahendra et al. [21]. However, these results differ from those of a study by Afacan et al. (2020), where it was observed that calprotectin levels in GCF increased following treatment. The authors suggested that this increase may be due to calprotectin's potential role in supporting healing post-treatment, owing to its antimicrobial properties [22].

The presence of calprotectin in saliva has also been shown in various studies [2,23,24]. Salivary calprotectin has been shown to be increased in periodontal diseases as well.

It was found in a study by Ramseier et al. in 2009 [25] that the calprotectin concentration in whole saliva was higher in severe periodontitis patients when compared to healthy individuals (p = 0.023). The increase may be due to the enhanced levels of inflammatory mediators repeatedly entering into whole saliva from GCF during gingival or periodontal inflammation, or it may be also due to the fact that inflammation can exert influence on the salivary glands as well. Moreover, bacteria within dental plaque might repeatedly trigger the salivary glands. Panov et al. in 2014 [2] studied how azithromycin has an effect on salivary calprotectin levels in individuals with periodontal disease and found that levels of calprotectin saliva reduced during treatment. A clinical trial by Kamatham and Chava in 2022 [26] concluded that calprotectin levels in saliva were reduced after the adjunct use of low-level laser therapy with scaling and root planing in patients with periodontitis.

There is less evidence in the existing literature, assessing the influence of NSPT involving scaling and root planing alone, on salivary calprotectin in chronic moderate periodontitis subjects. From the present study, it was found that the mean concentration of calprotectin in saliva at baseline in the periodontitis group was 0.17 μg/μl and three months following NSPT was 0.10 μg/μl. There was a decrease in the salivary calprotectin levels after therapy, but the difference from baseline is not statistically significant. It may be speculated that there might have been a flow of GCF into saliva due to its enhanced flow in the course of inflammation which may have affected the levels, hence providing information about the periodontal condition, but its production from the salivary glands, mucosal and carious lesions cannot be ruled out [27], which may be considered as one of the limitations.

Sun et al. in 2011 [28] were the first to recognize the association between periodontitis and enhanced calprotectin levels in plasma. In his study on subjects having aggressive periodontitis, compared to the controls, plasma calprotectin concentration in the case group was higher than the controls with statistical significance. A study by Haririan et al. in 2016 [29] demonstrated that serum and saliva calprotectin are elevated in patients with periodontitis. Since no study comparing serum levels of calprotectin after NSPT in chronic periodontitis subjects could be identified in the existing literature, an exact comparison with other studies cannot be done. In this study, the mean concentration of serum calprotectin of individuals with periodontitis before and three months following NSPT did not show any significant change. This may be attributed to the fact that serum components are affected by causes other than those related to periodontium and the inflammatory biomarkers may become diluted in serum compared to GCF and saliva [30].

The limitations of this study include, lack of microbiological analysis and its correlation with calprotectin level, possible laboratory errors due to manual method of estimation, small sample size, lack of proper method of assessment of the systemic status (relied only on patients’ history), lack of comparison of calprotectin levels with other inflammatory biomarkers, and confounding factors such as race/ethnicity was not adjusted. Moreover, significant differences in the mean age between the two groups may have influenced the study outcomes and could potentially introduce bias or confounding effects. Therefore, we consider this as another limitation of this study.

## Conclusions

Taking into account the study limitations, we can arrive at the conclusion that calprotectin concentration in GCF can be used as a potential measure to distinguish subjects with periodontitis from those without periodontitis since calprotectin concentration in GCF was elevated in periodontitis subjects in comparison to subjects without periodontitis.

Moreover, calprotectin can be considered as a biomarker that can be utilized to assess the treatment response, since its concentration is significantly reduced after treatment.

However, future studies and clinical trials using better technique-sensitive methods for the estimation of calprotectin levels needs to be carried out. Calprotectin concentration in serum and saliva may not be considered a good marker for assessing response to treatment.

## References

[1] Newman M, Carranza F, Takei H, Klokkevold P (2006). Carranza's clinical periodontology, tenth edition.

[2] Panov VE, Krasteva A, Krasteva AZ, Ivanova A, Panov A, Krastev Z (2014). Azithromycin decrease saliva calprotectin in patients with periodontal diseases. J IMAB.

[3] Andersen E, Dessaix IM, Perneger T, Mombelli A (2010). Myeloid-related protein (MRP8/14) expression in gingival crevice fluid in periodontal health and disease and after treatment. J Periodontal Res.

[4] Kido J, Nishikawa S, Ishida H, Yamashita K, Kitamura S, Kohri K, Nagata T (1997). Identification of calprotectin, a calcium binding leukocyte protein, in human dental calculus matrix. J Periodontal Res.

[5] Poullis A, Foster R, Mendall MA, Fagerhol MK (2003). Emerging role of calprotectin in gastroenterology. J Gastroenterol Hepatol.

[6] Hammer HB, Odegard S, Fagerhol MK (2007). Calprotectin (a major leucocyte protein) is strongly and independently correlated with joint inflammation and damage in rheumatoid arthritis. Ann Rheum Dis.

[7] Sander J, Fagerhol MK, Bakken JS, Dale I (1984). Plasma levels of the leucocyte L1 protein in febrile conditions: relation to aetiology, number of leucocytes in blood, blood sedimentation reaction and C-reactive protein. Scand J Clin Lab Invest.

[8] Kojima T, Andersen E, Sanchez JC, Wilkins MR, Hochstrasser DF, Pralong WF, Cimasoni G (2000). Human gingival crevicular fluid contains MRP8 (S100A8) and MRP14 (S100A9), two calcium-binding proteins of the S100 family. J Dent Res.

[9] Kido J, Nakamura T, Kido R, Ohishi K, Yamauchi N, Kataoka M, Nagata T (1999). Calprotectin in gingival crevicular fluid correlates with clinical and biochemical markers of periodontal disease. J Clin Periodontol.

[10] Lundy F, Chalk R, Lamey P, Shaw C, Linden G (2001). Quantitative analysis of MRP-8 in gingival crevicular fluid in periodontal health and disease using microbore HPLC. J Clin Periodontol.

[11] Kaner D, Bernimoulin JP, Kleber BM, Heizmann WR, Friedmann A (2006). Gingival crevicular fluid levels of calprotectin and myeloperoxidase during therapy for generalized aggressive periodontitis. J Periodontal Res.

[12] Nakamura T, Kido J, Kido R, Ohishi K, Yamauchi N, Kataoka M, Nagata T (2000). The association of calprotectin level in gingival crevicular fluid with gingival index and the activities of collagenase and aspartate aminotransferase in adult periodontitis patients. J Periodontol.

[13] Giannopoulou C, Andersen E, Brochut P, Plagnat D, Mombelli A (2006). Enamel matrix derivative and systemic antibiotics as adjuncts to non-surgical periodontal treatment: biologic response. J Periodontol.

[14] GR JC, VE JR (1964). The simplified oral hygiene index. J Am Dent Assoc.

[15] LO H, SI J (1963). Periodontal disease in pregnancy. I. Prevalence and severity. Acta Odontol Scand.

[16] Taba M Jr, Kinney J, Kim AS, Giannobile WV (2005). Diagnostic biomarkers for oral and periodontal diseases. Dent Clin North Am.

[17] Kido J, Nakamura T, Kido R, Ohishi K, Yamauchi N, Kataoka M, Nagata T (1999). Calprotectin in gingival crevicular fluid correlates with clinical and biochemical markers of periodontal disease. J Clin Periodontol.

[18] Badersten A, Nilvéus R, Egelberg J (1985). Effect of nonsurgical periodontal therapy. VII. Bleeding, suppuration and probing depth in sites with probing attachment loss. J Clin Periodontol.

[19] Obeid PR, D'Hoore W, Bercy P (2004). Comparative clinical responses related to the use of various periodontal instrumentation. J Clin Periodontol.

[20] Gao H, Xu J, He L, Meng H, Hou J (2021). Calprotectin levels in gingival crevicular fluid and serum of patients with chronic periodontitis and type 2 diabetes mellitus before and after initial periodontal therapy. J Periodontal Res.

[21] Mahendra J, Muralidharan J, Srinivasan S (2024). Calprotectin and periostin levels in periodontitis patients with coronary artery disease. Oral Dis.

[22] Afacan B, Çınarcık S, Gürkan A (2020). Full-mouth disinfection effects on gingival fluid calprotectin, osteocalcin, and N-telopeptide of Type I collagen in severe periodontitis. J Periodontol.

[23] Kleinegger CL, Stoeckel DC, Kurago ZB (2001). A comparison of salivary calprotectin levels in subjects with and without oral candidiasis. Oral Surg Oral Med Oral Pathol Oral Radiol Endod.

[24] Zhou M, Meng HX, Zhao YB, Chen ZB (2012). Changes of four proinflammatory proteins in whole saliva during experimental gingivitis. Chin J Dent Res.

[25] Ramseier CA, Kinney JS, Herr AE (2009). Identification of pathogen and host-response markers correlated with periodontal disease. J Periodontol.

[26] Kamatham SA, Chava VK (2022). Comparison of salivary calprotectin levels in periodontitis associated with diabetes mellitus after low-level laser therapy as an adjunct to scaling and root planing: A randomized clinical trial. J Indian Soc Periodontol.

[27] Holmström SB, Lira-Junior R, Zwicker S (2019). MMP-12 and S100s in saliva reflect different aspects of periodontal inflammation. Cytokine.

[28] Sun X, Meng H, Shi D (2011). Analysis of plasma calprotectin and polymorphisms of S100A8 in patients with aggressive periodontitis. J Periodontal Res.

[29] Haririan H, Andrukhov O, Pablik E, Neuhofer M, Moritz A, Rausch-Fan X (2016). Comparative analysis of calcium-binding myeloid-related protein-8/14 in saliva and serum of patients with periodontitis and healthy individuals. J Periodontol.

[30] Lira-Junior R, Öztürk VÖ, Emingil G, Bostanci N, Boström EA (2017). Salivary and serum markers related to innate immunity in generalized aggressive periodontitis. J Periodontol.

